# A conceptual model for worksite intelligent physical exercise training - IPET - intervention for decreasing life style health risk indicators among employees: a randomized controlled trial

**DOI:** 10.1186/1471-2458-14-652

**Published:** 2014-06-26

**Authors:** Gisela Sjøgaard, Just Bendix Justesen, Mike Murray, Tina Dalager, Karen Søgaard

**Affiliations:** 1 Institute of Sport Science and Clinical Biomechanics, University of Southern University, Campusvej 55, DK 5230 Odense, Denmark

## Abstract

**Background:**

Health promotion at the work site in terms of physical activity has proven positive effects but optimization of relevant exercise training protocols and implementation for high adherence are still scanty.

**Methods/Design:**

The aim of this paper is to present a study protocol with a conceptual model for planning the optimal individually tailored physical exercise training for each worker based on individual health check, existing guidelines and state of the art sports science training recommendations in the broad categories of cardiorespiratory fitness, muscle strength in specific body parts, and functional training including balance training. The hypotheses of this research are that individually tailored worksite-based intelligent physical exercise training, IPET, among workers with inactive job categories will: 1) Improve cardiorespiratory fitness and/or individual health risk indicators, 2) Improve muscle strength and decrease musculoskeletal disorders, 3) Succeed in regular adherence to worksite and leisure physical activity training, and 3) Reduce sickness absence and productivity losses (presenteeism) in office workers. The present RCT study enrolled almost 400 employees with sedentary jobs in the private as well as public sectors. The training interventions last 2 years with measures at baseline as well as one and two years follow-up.

**Discussion:**

If proven effective, the intelligent physical exercise training scheduled as well as the information for its practical implementation can provide meaningful scientifically based information for public health policy.

**Trial Registration:**

ClinicalTrials.gov, number: NCT01366950.

## Background

Physical activity during work and leisure is often the greatest stress that the body encounters in the course of daily life and calls for a number of physiological regulatory processes and their interplay which are entirely dependent on the type of the physical activity performed. For decades an extensive literature has considered physical activity to provide health benefits irrespective of the type or the site of physical activity performed. Typically, physical activity is divided into the domains of work and leisure, but the international recommendations for health-promoting physical activity do not distinguish between occupational and leisure time physical activity [1]. In this context there has been a lack of attention to the extensive literature documenting high intensity occupational physical activity to deteriorate health [2].

### Contrasting physical activity at work and leisure

A health paradox was recently addressed and data from the Danish Work Environment Cohort Study, DWECS, demonstrated long term sickness absence to decrease with increased leisure time physical activity but to increase with increased occupational physical activity [3]. Scrutinizing the patterns of physical activity in these two domains encountered major differences.

**Figure 1:**
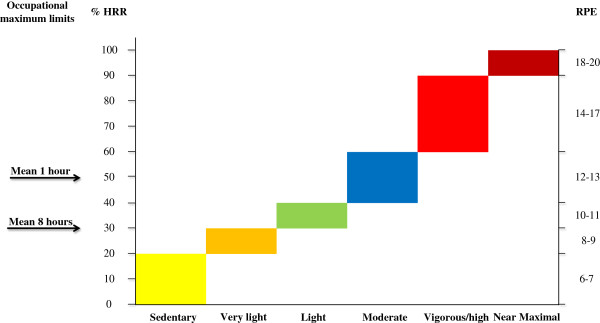
**Relationship between intensity of physical activity and the corresponding ranges for HR and RPE.** Exercise intensity terminology and corresponding HR and RPE intervals are presented in accordance with a compromise of two position statements [1,4] and the two arrows for maximum permissible occupational load for 8 hour work day and 1 hr work, respectively, are referring to [8].

According to the international recommendations on physical activity for health [1] it is recommended that: “most adults engage in moderate-intensity cardio-respiratory exercise for ≥30 min∙d^−1^ on ≥5 d∙wk^−1^ for a total of  ≥ 150 min∙wk^−1^, vigorous-intensity cardiorespiratory exercise training for ≥20 min∙d^−1^ on ≥ 3 d∙wk^−1^ (≥75 min∙wk^−1^), or a combination of moderate- and vigorous-intensity exercise to achieve a total energy expenditure of ≥500-1000 MET ∙min∙wk^−1^. On 2–3 d∙wk^−1^, adults should also perform resistance exercise for each of the major muscle groups, and neuromotor exercise involving balance, agility, and coordination”. The latter modes of training may be categorized as functional training. The target levels for physical activity in terms of duration and intensity for the cardiorespiratory exercise training can be obtained by accumulating smaller periods of physical activity, for example by splitting 30 min in 3 bouts lasting 10 minutes or more. Physical activity intensity levels have been divided into e.g. 6 categories (Figure 1) and related to %HRmax, heart rate reserve (HRR), % VO2max, metabolic equivalent task (MET), or rating of perceived exertion (RPE) for setting the framework for recommendation for exercise training to conquer life style diseases due to physical inactivity [1,4]. Such levels of recommended physical activity may have occurred in the cohorts of longshoremen [5] and bus-conductors [6] with data obtained at the end of the 19^th^ and beginning of the 20^th^ century. These early studies are cornerstones in our understanding of the relationship between physical activity and cardiovascular diseases and show positive relations between physical activity at work and cardiovascular health. However, the labor market has changed dramatically and work tasks performed with large muscle groups in terms of major dynamic physical activity are almost extinct. Accordingly, a more recent paper on the perspectives from these earlier studies stated that leisure time physical activities have to be included, “presumably because of lack of variability in intensities of physical work” [7].

The occupational physical activity in contrast to leisure time physical activity has to be endured up to 8 hours a day, 5 days a week, which in the middle of the 20^th^ century set fundamentally different levels compared with the health enhancing levels recommended [1]. Instead of minimum levels of physical activity, levels of maximum permissible intensity were proposed. Consensus guidelines were first time presented by the International Labor Organization in 1971 [8]. The maximum level of work intensity for an 8 hrs workday was a mean value of 30% of heart rate reserve estimated as delta value from resting HR to HRmax and is termed HRR as marked in Figure 1. Somewhat higher values of 50% HRR were acceptable if the work tasks have to be endured for only one hour a day. These intensities are only marginally reaching levels that will improve cardio-respiratory fitness. To this adds that over the last century the physical activity in many job types has steadily decreased [9] and a major concern for public health in modern working life is the aspect of inactivity; in part due to the sedentary working conditions. However, in spite of technical improvements and an increased computerization of job task many sectors still demand manual work in terms of continuous walking, standing, lifting, pushing, and pulling. These demands are especially common in work with personal care, heavy industry or service jobs but with great differences in the daily exposure profiles. An 8 hour work day with standing and walking such as cleaning may easily exceed the recommended guidelines but still not reach a level that could be health enhancing [10]. Similarly, jobs with awkward postures such as among health care workers or constructions workers with occasionally high peak loads may pose high demands on the low back but without the timing and intensity that could provide a training effect in general or specifically on the low back muscles. Optimal health enhancing training for the worker may depend on the occupational load - ranging from inactive or low to moderate and high mechanical and or metabolic loads- in combination with individual health and physiological capacity profiles.

### The work site as arena for health promotion

For a large part of the population the majority of the waking hours a day are spent at work and work has a major impact on our physical, mental, economic and social well-being [11]. Accordingly, WHO has pointed out the work place as a specially prioritized arena for public health enhancement, and has implied an almost ethical obligation for a commitment from the workplace. Work related disorders have been defined by WHO as multifactorial and include disorders where work significantly contributes to development or increase in pain and disorder [12]. Therefore, no clear border exists between the prevention and rehabilitation, and health enhancing initiatives should be a naturally integrated part of the general improvement and maintenance of work environment aiming both at employees with health problems as well as healthy workers. A decrease in health status - whether caused by work or not - may influence the worker’s productivity and work ability [13]. Thus, companies have an economic interest in health enhancing interventions, and in addition they constitute an organization and infrastructure that may be ideal for interventions tailored to specific groups in the population.

### Evidence of health enhancing physical activity at the work site

During the last decade an increasing number of randomized controlled trials, RCT, have been conducted introducing physical exercise training programs at the worksite often performed during working hours. Growing evidence is presented that such programs result in clinically relevant health effects and preliminary cost effectiveness estimates indicate acceptable cost relative to societal savings on health expenses [14,15]. Work site health promotion and job exposure dependent exercise training is thus contemporarily recognized as a significant tool for health improvement in the workforce and thereby also in benefit of the company. Complementing the work exposure profile to develop a healthy exposure/training profile by integrating leisure time activity in work life is increasingly considered cost effective. In Denmark we have recently conducted nine RCT’s successfully in terms of improving health among a number of different job categories ranging from physically inactive to low, moderate, and finally, heavy physical work. The interventions enrolled ~ 2500 workers and lasted from 10 – 52 weeks. Questionnaire surveys and health checks were performed at baseline and follow-up. The job groups included were: Office or computer workers [16-18], industrial laboratory technicians [19], cleaning personnel [20], health care workers [21], construction workers [22], and fighter pilots [23]. Relative aerobic capacity –a health risk indicator for cardio-vascular diseases- was improved among office and computer workers, health care workers, and construction workers while a reduction in musculoskeletal disorders – a main cause for sickness absence – was seen in office and computer workers, industrial laboratory technicians, cleaning personnel as well as fighter pilots, and a number of other improvements in physical capacities were evidenced with effect sizes of clinical relevance.

Three essential factors characterized these interventions which made them distinct from a number of unsuccessful interventions: 1) Physical exercise training was performed during working hours 1 hr∙wk^−1^, usually divided into 2–3 training sessions, which requested involvement of the employer to allow for such activities and thus signaling support of health enhancement for employees, 2) sports exercise training specialists were involved in designing the specific exercise training programs that were evidence based and of generally high intensity, 3) training sessions were regularly supervised by expert trainees in the field and adherence was monitored. It is concluded that worksite exercise training does enhance health if a program with evidenced efficacy is implemented by expert trainees with support of the employer. In these studies the training programs were similar for all participants, however, from a highest benefit perspective training should be individually tailored.

### Adherence to physical exercise training

Although the RCT’s mentioned above were successful in improving health, their effect sizes could be improved by increased adherence to the training by the enrolled employees. Regular adherence was as a mean 61% but among the studies ranged from 31% to 86%, which allows for significant improvement in most studies. The implementation of the training needs more attention since this may improve adherence and thereby the effect size [24]. This is true for exercise programs offered during working hours and in particular for motivation of adequate exercise training during leisure. The latter may be particularly crucial in job categories characterized by major inactivity, since the total duration of physical activity requested in a health perspective amounts to several hours a week, which every employer may not accept as working time. Such leisure physical activities may be planned in a social context at the company and attended just before or after working hours. Thus the worksite may be considered as an ideal arena for implementation of physical activity: it is organized with information and communication systems, has contact with its workers on a daily basis, and may take social responsibilities. These features may play significant roles in scheduling physical exercise training for the worker during working hours, in relation to work time – i.e. before or after, or during leisure by announcing relevant info from around the local community on these issues.

### Cost-effectiveness

Preliminary cost effectiveness estimates in worksite RCT’s conducted in Denmark indicate acceptable cost relative to societal savings on health expenses. However, more subtle analyses are requested. The present study therefore will record relevant information for cost-effectiveness estimates. While focus primarily has been on the cost of sickness absence that with some uncertainties can be calculated, a number of new studies point towards the much larger but more invisible cost of employees with a health related reduced productivity and work ability but still maintaining work [25]. This so-called “presenteeism” may economically be a much more important factor to consider for the companies [13]. In the present study in addition to absenteeism measured as sickness absence, also presenteeism is evaluated based on self-reported productivity [26] and work ability [27].

**Figure 2:**
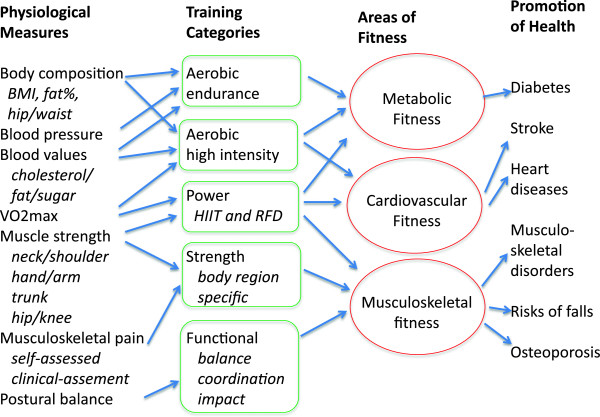
**Physiological assessment variables as premise for recommendation of IPET for a sustained healthy work force.** RFD: rate of force development and HIIT: high intensity interval training.

The aim of this paper is to present a study protocol with a conceptual model for planning individually tailored physical exercise training for each worker, optimized by the use of an individual health check, existing guidelines and state of the art sports science training recommendations in the broad categories of cardio-respiratory fitness, muscle strength in specific body parts, and functional training including balance training (Table 1). The cartoon in Figure 2 specifies health check variables and how they point towards various beneficial training categories for improving areas of fitness that are evidenced for promotion of specific health benefits. The areas of fitness were aligned with those in a recent review in the area to consult for relevant references [28]. Special attention was given to the procedure of implementing the training in order to optimize adherence.

**Table 1:** Outcome measures from the health check and questionnaire for selecting optimal individually tailored training programs within 5 different training modes

**Health measures**	**Cardio**	**Strength**		**Function**	
	**Moderate to high intensity**	**Neck and shoulders**	**Large muscle groups**	**Core stability**	**Balance**
**Questionnaire**					
Symptoms neck/shoulder		X			
Symptoms lower back				X	
**Strength test**					
Strength neck/shoulder		X			
Strength back/abdominal			X		
**Chiropractor check**					
Core stability				X	
Neck/shoulder stability		X			
**Physiological health check**					
Aerobic fitness test	X				
Body mass index (BMI)	X				
Body fat%	X				
Blood pressure	X				
Blood fat (LDL + HDL)	X				
Blood glucose	X				
Balance test					X

The hypotheses of this research are that individually tailored worksite-based intelligent physical exercise training, IPET, among workers with inactive job categories will:

1. Improve cardiorespiratory fitness and/or individual health risk indicators

2. Improve muscle strength and decrease musculoskeletal disorders

3. Succeed in regular adherence to worksite and leisure physical activity training

4. Reduce sickness absence and productivity losses (presenteeism) in office workers

## Methods and design

### Study design

The study is a prospective two years parallel group, examiner-blinded, randomized controlled trial with an intelligent physical exercise training (IPET) intervention group and a control group (CG) conducted in accordance with the CONSORT statement [29]. The enrollment was sequential in 6 strata from May 2011 to March 2012 and with baseline as well as one and two year follow-up measures. All participants gave their written informed consent to participate. The local Ethics Committee of Southern Denmark approved the study protocol (S-20110051), which qualified for registration in ClinicalTrials.gov (NCT01366950). Employees were assigned an arbitrary ID number, which was concealed by an authorized technical staff person. The IDs were randomized individually within each strata following baseline measures using a random number computer algorithm and balanced for gender in strata with less than 100 employees. All test personnel and investigators involved in data treatment were blinded to the randomization.

### Study population

**Figure 3:**
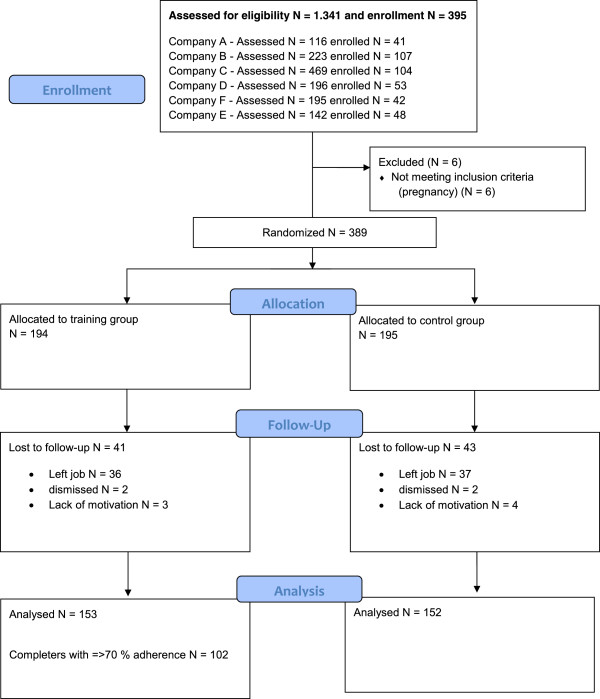
Flow chart of recruitment of employees.

In total, 103 companies all over Denmark were contacted by e-mail in May 2010 based on individual business relations to contact persons in these companies. Seventeen of these companies were interested in receiving more information, and these were visited with meetings where researchers presented the project to the contact person (representing employees) and representatives from the top management level. Before the deadline for inclusion (March 2011) 6 companies agreed to participate, while another 10 companies wanted to join the project later but were not included in this study. The companies enrolled comprised two private companies, two public municipalities, and two national boards (three companies in Jutland (Western Denmark), one in Funen (mid Denmark), and two in Zealand (Eastern Denmark)). Strata and enrollment dates: Company A (private company 1) May 2011, Company B (municipality 1) June 2011, Company C (municipality 2) December 2011, Company D (national board 1) January 2012, Company E (national board 2) January 2012, and Company F (private company 2) March 2012. See flow chart in Figure 3.

Inclusion criteria were employment as office worker working at least 25 hours a week. Exclusion criteria were pregnancy and severe musculoskeletal disorders or other severe health issues such as cardiovascular diseases (e.g. chest pain during physical exercise, myocardial infarction and stroke), symptomatic herniated disc or severe disorder of the spine, postoperative condition or history of severe trauma.

### Procedure

Employees as well as top and middle management at all 6 companies were informed about the project via intranets and a date for an information meeting was announced 2 months in advance. Further, the contact person at each company announced the information meeting to all top and middle managers by e-mail together with the president for Human Resource. Information meetings addressed the overall aim of the study as well as practicalities such as: type of physical exercise programs, site of training, health check, instructors, and health ambassadors. All were offered information about the project in paper copy, and could ask questions. The information was subsequently placed at the intranet of each company accessible for everybody at the company. Shortly after the information meetings all employees were invited and received an electronic questionnaire. Completing the questionnaire was a prerequisite for becoming part of the project.

#### Health ambassadors

Among the eligible employees, peers were appointed to act as change agents and termed health ambassadors. Their job was to motivate colleagues in the training group to become and sustain physically active during the course of the research project. A health ambassador was appointed for every 10 – 15 employees in the department joining the intervention group by selection, and should be willing to work with health aspects related to the project at the workplace for 2 hours a week for two years. The health ambassadors were part of the training group but were not part of the randomizing and were excluded from analysis because of selection bias as they are a part of the implementation process. The health ambassadors completed a 4 day course before the intervention started dealing with the themes: Health enhancing physical activity – evidence, myth and gains; ethical issues; theories for changing behavior; cataloguing ideas for practical facilities; organization, motivation and communication [30].

In order to select employees to take the role of health ambassadors the middle managers in the 6 companies were asked to identify and appoint candidates in their department using all following criteria: A health ambassador should be a team worker, find it easy to motivate colleagues, enterprising at nature, and having at least 5 years seniority at the workplace. The potential health ambassadors were asked by their middle manager to join the project allowing them the possibility to decline.

#### Instructors

Instructors were appointed among bachelor students in the education of sport and health at the University of Southern Denmark. Prior to the intervention instructors were informed about the project and their role in the project. The instructors had the following job description: Make sure employees in the training groups complete all exercises described in their protocols and supervise all employees to train at high intensity and with the proper techniques. Training intensity was assessed by the Borg scale (RPE 6–20) by each participant at the end of each training session. Target training intensity was 14–17 RPE for every training session and RPE together with training attendance were registered by the instructor.

#### Health check

Participants had scheduled a one hour health check at baseline before the intervention and at follow up after one and two years of intervention. Measurements included were: VO_2max_, muscle strength, body mass, body height, body fat percent, waist/hip ratio, blood pressure, blood lipid and glucose profile. Additionally a balance test was performed. All employees received individual notification shortly after the checks. Cut-points for recommending specific training modes are given in the section “Outcome measures”, see also Table 1.

#### Musculoskeletal check

An objective examination was performed by a chiropractor for musculoskeletal disorders at baseline before the intervention lasting approximately ½ hour. Based on the musculoskeletal check employees were excluded (see exclusion criteria above) if considered in risk of deteriorating health by a major part of the training program or they were advised with individual recommendation to take special care in relation to specific exercises e.g. in case of minor musculoskeletal disorders. The musculoskeletal check consisted of a body position test [31,32], movement test [33-35], stability-palpation test [36,37]. Based on this check the chiropractor could recommend training of core stability or neck/shoulder strength (see Table 1).

### The intervention program

A conceptual model was developed for designing individually tailored programs termed “Intelligent Physical Exercise Training”, IPET. The concept of IPET at the worksite was: 1) to balance the physiological capacity of the employees relative to occupational exposure, 2) to tailor the exercise to individual capacities and disorders to improve employees’ health, 3) to motivate participants by offering evidenced and enjoyable programs implemented with care, and 4) to be cost-effective for the company.

**Ad 1)** The occupational exposure was sedentary work with intense computer use for all eligible participants in this study. This implied that inactivity had to be reduced by following the guidelines from American College of Sports Medicine [1]. To reach these targets in quantity it was negotiated with employees and top-management that 1 hr of vigorous intensity training (77 – 95%HRmax, RPE 14–17) per week would be performed during working hours and 30 min moderate intensity training (64–76%HRmax, RPE 12–13) six days a week during leisure by each participant or a minimum of 3 hrs per week. The instructors were supervising the 1 hr vigorous intensity training at facilities in the workplace or in the local area one day a week during the first year; during the second year they supervised only 1 hr a month. The health ambassadors were active in advising and motivating for the leisure time training, mainly dynamic activities using large muscle groups (64–76%HRmax)

**Ad 2)** The health check and musculoskeletal check as well as questionnaire served to tailor the activities to the individual for the high intensity training 1 hr a week at the worksite. The training was composed of the 3 major training categories: Cardio-respiratory fitness, strength training, and functional training (or neuromuscular training). The latter two were subdivided into strength training for neck/shoulder or large muscle groups, and functional training for core stability or balance, respectively, resulting in 5 different categories to allocate training duration for (see Table 1). Specific cut-points were set from the health check measures for recommending a specific training category (see below). Additionally, questionnaire reply (see below) of musculoskeletal complaints for > 30 days/year in neck/shoulder or low back resulted in recommending training of neck/shoulder strength and functional core stability training, respectively. Likewise, the musculoskeletal check could result in recommended training of neck/shoulder strength and functional training of torso and/or balance. Depending on the number of recommended training categories (max 5) the time allotted to these exercises was adjusted according to Table 2. Several cut-points often qualified for recommending the same training category, but the time allocated to the five different training categories was independent of the number of tests surpassing a cut-point.

**Ad 3)** Instructors as well as health ambassadors were recruited to assist the participants and spur their motivation (see above). Further, each employee received a hand-out with an individual intelligent physical exercise training program. All training sessions lasted 50 min – allowing 10 min for getting to and from the site of work and the site of training within the one hour off work – and were led by instructors. Of these sessions always 20 min were in the beginning allocated to cardio-respiratory fitness training, including 10 min warm-up, due to the physical inactivity at the job. After this the instructor guided each employee to train their specific exercises and at recommended training intensities. Those employees who did not surpass any of the cut-points for recommending specific training categories were to train 25 min cardio-respiratory fitness and 25 min strength training for large muscle groups while all others had specifically composed training categories (Table 2).

Exercises for cardio-respiratory training were up to the employee to choose with guidance from instructors and with the focus on training at high intensity and could be running, stepping, rowing, ball games etc. Exercises for strength training of large muscle groups were selected from 5 different exercises: 1 for shoulders, 3 for abdomen-back and 1 for the breast muscles. The intensity for strength training of large muscle groups was 60 – 80% of one repetition maximum, RM. Frequency: 3 sets of 8 repetitions. Breaks: Employees were instructed to shift between exercises, which meant maximum 10 s breaks between sets. Exercises for neck and shoulder strength were shrugs, reverse flyers, and arm abduction, i.e. evidenced previously to increase strength/endurance and reduce neck/shoulder pain [17,38]. The intensity for neck and shoulder training was to pain limits or as heavy as possible and with proper technical execution. Frequency: 3 sets of 8 repetitions. Breaks: 1 – 2 min breaks between sets. Exercises for functional training were selected from 9 different exercises: 5 for balance training and 4 for core stability training. Functional training had no demands for intensity or frequency.

**Ad 4)** Measures of sickness absence recorded by the company and responded to the questionnaire by the employer together with their data on salary and self-reported productivity and workability will feed into models of health economics [14]. Account will be taken on the hours of training during working hours.

**Table 2:** **All possible combinations of the five training categories in Table **1** and the time (minutes) allotted to each category for each combination**

**Number of exercise categories**	Combination of exercise categories (*)	**Cardio (Ca)**	**Extra cardio**	**Neck/Shoulder (NS)**	**Large Muscles (LM)**	**Core Stability (CS)**	**Balance (Ba)**	**Combi- nations used**
**0**		20	5		25			
**1**	**Ca**	20	5		25			21
	**NS**	20		20	10			1
	**LM**	20	5		25			
	**CS**	20	5		5	20		
	**Ba**	20	5		20		5	
**2**	**Ca + NS**	20	5	20	5			17
	**Ca + LM**	20	5		25			
	**Ca + CS**	20	5		5	20		2
	**Ca + Ba**	20	5		20		5	
	**NS + LM**	20		20	10			3
	**NS + CS**	20		20		10		12
	**NS + Ba**	20		20	5		5	1
	**LM + CS**	20			15	15		
	**LM + Ba**	20	5		20		5	
	**CS + Ba**	20			10	15	5	
**3**	**Ca + NS + LM**	20		20	10			17
	**Ca + NS + CS**	20		20		10		33
	**Ca + NS + Ba**	20	5	20			5	6
	**Ca + LM + CS**	20	5		15	10		16
	**Ca + LM + Ba**	20	5		20		5	3
	**Ca + CS + Ba**	20	5			20	5	1
	**NS + LM + CS**	20		15	5	10		16
	**NS + LM + Ba**	20		15	10		5	
	**NS + CS + Ba**	20		15		10	5	7
	**LM + CS + Ba**	20			15	10	5	
**4**	**Ca + NS + LM + CS**	20		15	10	5		18
	**Ca + NS + LM + Ba**	20		15	10		5	1
	**Ca + NS + CS + Ba**	20	5	15		5	5	15
	**Ca + LM + CS + Ba**	20	5		10	10	5	1
	**NS + LM + CS + Ba**	20		15	5	5	5	
**5**	**Ca + NS + LM + CS + Ba**	20		10	10	5	5	3

(*) Ca: Cardio training, NS: Neck/shoulder strength training, LM: Large muscle groups strength training, CS: Core stability functional training, and Ba: Balance functional training.

Last column shows the number of participants who were allocated to the combination at baseline.

### Outcome measures

#### Objective measures

Measures obtained during the health check were:

##### Estimated maximal oxygen uptake (VO_2max_)

VO2 max was the primary outcome for this study and was estimated from the relation between sub-maximal workload and steady state heart rate obtained in Åstrand one-point sub-max test using the Åstrand nomogram [39] and correcting for age [40]. The tests were performed on a bicycle (Monark 874E, Monarch Exercise AB, Sweden) and with heart rate (HR) measured (Polar S610i Heart Rate Monitor and Polar FT2 Heart Rate Monitor). Test procedure: The starting load was 60 Watt for women and 90 Watt for men pedaling at 60 rpm. After 2 min of warm-up the load was adjusted based on the measured HR. If the HR was below 120 beats per min (bpm) the load was adjusted with 30 Watt every minute until a steady state HR (change of ≤ 4 bpm per 1 min) was reached between 120 – 170 bpm. The test length was maximum 10 min and employees were instructed not to talk during the test. The cut-point for recommending extra cardio-respiratory fitness training was a test value <80% of the reference value from the Danish working population [41].

##### Muscle strength

Maximal isometric muscle strength was measured with Bofors MODEL dynamometer (Bofors Elektronik, Karlskoga Sweden) mounted in a reproducible standardized setup for 4 tests: Shoulder elevation, arm abduction, back extension, and abdominal flexion. In every test the employees completed 3 maximal voluntary contractions (MVC) with at least a 30 s break between tests (if the 3^rd^ MVC was 5% higher than the first and second test the employee was instructed to perform another test with the maximum of 5 tests). The highest value was recorded and moment arm for all test was registered [42]. The cut-point for recommending strength training was a test value for a particular body region <80% of the reference value from the Danish working population [41].

##### Balance test

A unilateral stance test was performed with eyes open and participants were instructed to look directly ahead at a black spot placed approximately 2 meters in front of them at eye height. The participants stood on the dominant foot (defined as the foot used for standing while kicking a ball) with the big toe of the non-dominant foot leaning against the medial malleolus of the dominant foot. The test was performed for 30 s [43]. Each participant was allowed three trials with loss of balance before the end of the test being classified as failed. The cut-point for being allocated to balance training was failure of all three trials.

##### BMI and body fat

Body height, weight, and fat were measured using a bio impedance device (Tanita TBF 300). Employees were normally hydrated and were measured without shoes and socks and with light clothing. Waist/hip ratio was measured with a ruler [44]. The cut-points for recommending cardio-respiratory training were: BMI ≥ 25 or fat% >24 to >44% depending on age and sex [45].

##### Blood pressure

Blood pressure was measured in seated position after 5 – 10 minutes of rest. Blood pressure was measured on the right arm with an electronic blood pressure device (OMRON M7). Blood pressure was measured 3 times with one minute rest between tests and the mean of the 2 lowest values was calculated [46]. The cut-point for recommending cardio-respiratory training was diastolic pressure > 90 mmHg or systolic pressure > 140 mmHg.

##### Blood profile

On the health check day overnight fasting blood samples (>7 hrs) were taken between 07:00 – 09:00 am. Blood samples were handled by technical personal from The University of Southern Denmark. The cut-points for recommending cardio-respiratory training were: Blood sugar ≤ 4 or ≥7 mmol/l, blood triglycerides ≤ 2 mmol/l, total cholesterol ≥6 mmol/l, LDL ≤ 3 mmol/l, and HDL ≥ 1 mmol/l.

#### Self-reported measures

##### Questionnaire

All employees completed a questionnaire three times: at baseline as well as after one and two years of intervention. Questions addressed: demographics, education, job, income before tax, productivity [26], workability [27], psychosocial aspects [47], pain/kinesiophobia, self-rated health [48], sick leave, smoking, alcohol, physical activity at leisure [49], and sports activities specified as: ball games, Nordic walking, jogging, cycling, aerobics, spinning, dancing, swimming, kayaking/rowing. Questions regarding musculoskeletal disorders were assessed by a modified version of the Nordic questionnaire of musculoskeletal disorders [50]. Cut points for recommending strength training for neck/shoulder or functional training for core stability were > 30 days complaints during the last year in the neck/shoulder area and low back area, respectively. At follow-up after one and two years, respectively, questions regarding adherence as well as the impact of the health ambassadors were included. Their impact was rated from 1 for no impact to 10 for major impact.

##### Diary

Employees in the training group reported every week to the health ambassador regarding their physical activity at leisure. The reports were categorized into: Running, organized fitness training, strength training, home & gardening, climbing stairs. The total time for activity as well as time with moderate and high intensity, respectively was inquired.

#### Report by instructor

The instructors filled in a training diary with attendances and intensity of training in terms of RPE for employees in the training group for each 1 hr weekly scheduled training sessions.

#### Company registered data

Each company delivered their records of sickness absence for all employees included in the study for three years: the year before baseline measures, as well as first and second year of intervention.

### Statistics

All results are reported as mean (SD) and p < 0.05 is considered statistically significant. Primary and secondary outcomes will be analyzed within (paired t-test) and between the intervention group and the control group after intervention (ANCOVA). Categorical variables will be tested using chi-square and McNemar tests. Analyses will be performed using SPSS statistical software, version 21. Intention to treat analyses will be performed and values carried forward and backwards for missing values in both baseline and follow up measurements. If measurements have missing values in both baseline and follow up they will be replaced by means of all existing data in each group, respectively. Per-protocol analysis will be carried out with employees from the training group who meet the criteria of at least 70% adherence in the training period [51]. Sample size calculation is based on a 5% improvement in VO_2max_ on a group level with a SD of 20%, type 1 error of 5% and a power of 80% which showed a requirement of 128 employees in each group [52]. With an estimated dropout of 30% the research project has to recruit around 400 employees.

## Discussion

In RCT studies it is a common strategy to give all participants in the intervention group the same treatment in absolute terms. In studies on physical activity for health promotion the exercises have been cardio-respiratory fitness training at given percentages of VO2max [53,54], strength training at a given percentage of maximum voluntary contraction [17] or fixed combinations [51]. The novelty of this study is that the exercise programs were individually tailored. This may enhance the individuals’ health promotion but at the same time impair the strength of the study in terms of significant effect on the primary outcome. A first attempt in developing IPET was among construction workers [22] where individually tailored exercise programs were developed based on a health check similar to the one in the present study. A combination of cardio-respiratory and strength training for specific body regions was developed for each individual employee and implemented for 12 weeks. A second step was to test intelligent physical exercise adjusted to the work place in terms of possible flexibility in duration, frequency, and supervision in order to fit the IPET to the work tasks [38]. Strength training was performed for 20 weeks but with highly variable training patterns. In the present study, optimizing the duration of the various exercise modes within the 1 hr training once per week during working hours was a challenge and we based our decisions on evidence based exercise training physiology (Table 2). The minimum of training for each of the five categories was 5 min. Further, we considered strength training with large muscle groups also to induce an effect on cardio-respiratory fitness. The last column in Table 2 shows the actual frequencies of the recommended training schedules in the present study. Of the in total 32 potentially possible combinations only 9 combinations were recommended for 12 or more of the participants, i.e. ≥3% of the total number of participants in each of these combinations. Interestingly, these 9 combinations together included more that 85% of all participants. This underlines that such large variety of training schedules as presented in Table 2 may not be needed. Actually, the last column also shows that around 3/4 of employees were recommended neck/shoulder strength training and a similar number cardio training. Both of these training categories were represented in 7 of the above 9 combinations. The particular need for these training activities are not surprising for this workforce, since they are sedentary 8 hrs five days a week and work in constrained neck/shoulder postures. Similarly, it is likely for other occupational groups based on health checks to identify optimal training combinations that are practically manageable at the work site.

### Strengths and limitations

A strength of this study is the rigid RCT design and the involvement of experts within occupational health as well as sports science. However, the enrollment of only approx. 30% of the eligible employees impairs the external validity. Also the randomization on an individual level may have introduced some contamination and cluster randomization could have been superior, but unfortunately not manageable in the present study due to the organization of the enrolled companies.

### Impact of results

The conceptual model presented here is easily applicable for practical use. If proven effective, the intelligent physical exercise training scheduled as well as the information for its practical implementation can provide meaningful scientifically based information for public health policy and health promotion strategies for employees in job groups at high risk for physical inactivity. This knowledge can be beneficial for occupational health professionals, supervisors, companies and employees in these job groups. Because the interventions are carried out during ordinary circumstances at a wide range of Danish workplaces, it is expected that the findings can be transferred and interventions implemented in other workplaces with high physical demands.

## Competing interests

The authors declare that they have no competing interests.

## Authors’ contributions

GS and JBJ have made substantial contributions to conception and design, MM and TD have given conceptual contributions and carried out acquisition of data together with JBJ, and KS has contributed significantly with conceptual aspects. All authors have been involved in drafting the manuscript and revising it critically for important intellectual content; and have given final approval of the version to be published.

## Pre-publication history

The pre-publication history for this paper can be accessed here:

http://www.biomedcentral.com/1471-2458/14/652/prepub
